# Reviewing the evidence on case management: lessons for successful implementation

**DOI:** 10.5334/ijic.825

**Published:** 2011-12-20

**Authors:** Nick Goodwin

**Affiliations:** Editor-in-Chief, IJIC and Senior Fellow, The King's Fund, 11-13 Cavendish Square, London, W1G 0AN, UK. Phone +44 207 307 2539, E-mail: ngoodwin@kingsfund.org.uk

Case management is a key tool in the delivery of integrated care and has become an internationally accepted model of providing intensive care support to people with long-term and complex health and social care needs. It takes many forms, but primarily represents a targeted, community-based and pro-active approach to care that involves case finding, assessment, care planning, and care co-ordination [1].

Since the first edition of *IJIC* in October–December 2000, much of the research and case study material we have published has focused in one way or another on the ability of care systems to meet the needs of the growing numbers of older people, and the related numbers of people with complex and long-term care needs. Much of this body of work has examined variations on the case management approach, typically looking at how the development of multi-disciplinary teams can better co-ordinate and integrate services around the needs of patients and service users and/or help them navigate their way through the complex web of services they need. The goal of such approaches has been to improve care experiences and care outcomes whilst simultaneously seeking to reduce the utilisation of expensive facilities in hospitals and/or nursing homes.

The evidence for the impact of case management revealed in this body of work, however, is decidedly mixed. The problem appears to be that case management will not deliver better care for patients and produce cost savings unless it is well designed, involve appropriately and professionally trained case managers and teams, and be embedded in a wider system of care that supports and values integrated and co-ordinated care.

Such lessons have been described throughout the history of this Journal from its very first edition when Kodner and Kyriacou's [2] expert analysis of two American models—the Social Health Maintenance Organisation (HMO) and the programme for all-inclusive care for the elderly (PACE)—concluded that well-designed, intensive and longitudinal case management could be highly effective in supporting frail elders. In 2011, however, Lewis et al. [3] reported that the evidence for the effectiveness of home-based case management for older people remained very limited internationally in terms of its ability to reduce hospitalisations and costs of care.

From both these articles it is clear that case management can be successful, but only where a number of key design factors are included. A recent review of the evidence by the health policy research team at The King's Fund in the UK has listed those factors that appear to be particularly important [1]:

- Assigned accountability of an individual (such as a nurse) or team for the individual being case-managed in order to provide continuity in how patients access services but also ensure a single line of responsibility for the care and services that a person receives regardless of where in the system they are receiving care;
- Clarity about the role of the case managers and support to ensure they have the right clinical skills and managerial competencies;
- Accurate case finding to ensure interventions are targeting patients with defined care needs;
- Appropriate caseloads to ensure that patients are receiving optimal care;
- A single point of access for assessment and a joint care plan;
- Continuity of care to reduce the risk of an unplanned admission to hospital;
- Self-care, to empower patients to manage their own conditions and to not become dependent on the care system;
- Integrated health and social care teams delivering services jointly;
- Information systems that support communication, and data that is used pro-actively to drive quality improvements.

Perhaps most importantly, the evidence suggests that case management will not succeed unless it is part of a wider programme of care in which multiple strategies are employed to co-ordinate services [4]. This includes good access to an extended range of primary care services, investment in health promotion and primary prevention, and co-ordinating community-based packages of social care to enable rehabilitation, re-ablement and independent living. Given the poor levels of care co-ordination that currently exist in many care systems, leading to poor care experiences and fuelling unnecessary expenditure, it remains imperative that services be better integrated around the needs of individuals. Where designed well, case management will have an important part to play in this strategy.
